# Style of pictorial representation is shaped by intergroup contact

**DOI:** 10.1017/ehs.2019.8

**Published:** 2019-07-23

**Authors:** Carmen Granito, Jamie Tehrani, Jeremy Kendal, Thom Scott-Phillips

**Affiliations:** 1 Department of Anthropology, Durham University, Durham DH13LE, UK; 2 Department of Cognitive Science, Central European University, Budapest 1051, Hungary

**Keywords:** Cultural evolution, graphical communication, art, style, language evolution

## Abstract

Pictorial representation is a key human behaviour. Cultures around the world have made images to convey information about living kinds, objects and ideas for at least 75,000 years, in forms as diverse as cave paintings, religious icons and emojis. However, styles of pictorial representation vary greatly between cultures and historical periods. In particular, they can differ in figurativeness, i.e. varying from detailed depictions of subjects to stylised abstract forms. Here we show that pictorial styles can be shaped by intergroup contact. We use data from experimental microsocieties to show that drawings produced by groups in contact tended to become more figurative and transparent to outsiders, whereas in isolated groups drawings tended to become abstract and opaque. These results indicate that intergroup contact is likely to be an important factor in the cultural evolution of pictorial representation, because the need to communicate with outsiders ensures that some figurativeness is retained over time. We discuss the implications of this finding for understanding the history and anthropology of art, and the parallels with sociolinguistics and language evolution.

## Graphical abstract

**Figure:**
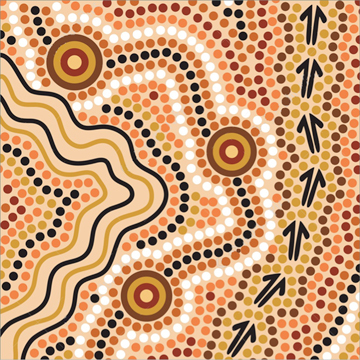

**Social media summary:** Cultures develop very different styles of pictorial representation across time and space. Why do they vary from largely recognisable figures of people and things to very abstract and opaque forms? One reason could be the presence or absence of intergroup contact. A study with experimental microsocieties shows how the need to communicate with outsiders can ensure that pictorial signs retain figurativeness over time.

## Introduction

Pictorial representations are ubiquitous in human culture. We find them in visual art, pictographic writing systems, road signs, graphic design, book illustrations, comics and animations, just to mention a few examples (Drucker and McVarish 2009; Harthan 1997; Hockney and Gayford 2016; Sabin 2001). Pictorial representations are tangible expressions of ideas, mental models and ways of understanding the world. They are highly versatile: they can visualise simple physical objects as well as very complex and abstract concepts and situations; as such, at the individual level, they are external cognitive tools that help elaborate, manipulate, store and retrieve ideas that would be difficult for the mind alone to handle, such as beliefs about supernatural agents (Mithen 1998, 2004, 2009). Pictorial representations are also effective attention-catching devices, especially when depictive and decorative techniques enhance their aesthetic appeal (Donald 2009; Gell 1992). They are sometimes easier to remember than words (Madigan 2014; Scaife and Rogers 1996) and, unlike spoken words, they are durable material objects that can reach different audiences and thereby influence minds and affect behaviours in different times and places (Donald 2006; Gell 1998). At the social level, pictorial representations are an effective tool of social coordination, a powerful means to disseminate ideas within a community, transmit them from generation to generation, and create shared worldviews; this makes them ideal vehicles to disseminate ideologies, both religious and secular (Collins 2016; Donald 2009; Mithen 2009). Humans have made use of pictorial representations since before the Upper Palaeolithic (Bahn 2016; Henshilwood *et al*. 2002), and image-making is likely to have played an important role in the evolution of human cognition and sociality (Renfrew and Morley 2009).

**Figure 1.**
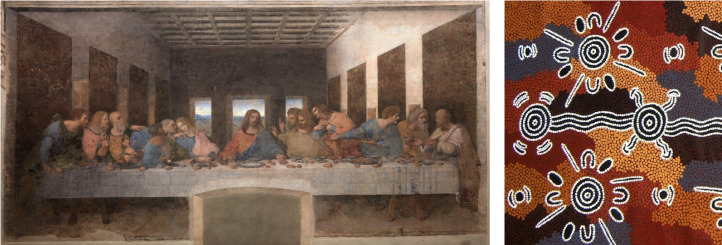
Examples of figurative and abstract style of representation. Leonardo da Vinci, *Last Supper* (1495–1498) and a piece of Warlpiri art (Australia). Both represent people sitting together, but they do so in radically different ways. The first uses figures (humans) that could be recognised potentially by any observer, whereas the second uses stylised shapes that are far less likely to be recognised as persons by observers not belonging to the Warlpiri community.

A central set of questions across several fields – in particular art history, anthropology, archaeology and the evolution of graphical communication systems – concerns the relation between styles of pictorial representation and characteristics of the social and demographic contexts in which they are produced (Boas 1927; Conkey and Hastorf 1990; Dressler and Robbins 1975; Fay *et al.* 2008; Fischer 1961; Garrod *et al.* 2007; Gombrich 1960; Healey *et al.* 2007; Lévi-Strauss 1962; Merrill 1987; Peregrine 2007; Schapiro 1994; Silver 1981). Pictorial representations vary across time and space in the strategies and conventions used to visualise things and ideas on a bi-dimensional surface. In particular, pictorial styles can greatly differ in their degrees of figurativeness, varying from largely inter-subjectively recognisable depictions of objects, people, animals and scenes, to very stylised and abstracted forms (Willats 1997; see Figure 1). Several cases have been observed where changes in figurativeness occurred in conjunction with contact between cultural groups, e.g. in Aboriginal art (Layton 1992b; Morphy 1991, 1998; Morphy and Layton 1981), Jewish art in medieval Germany (Shatzmiller 2013), Greco-Oriental art in the Hellenistic period (Versluys 2017) and Early Christian art in Syria (Verstegen 2012).

However, the empirical causality and generality of the relationship between changes in the nature and extent of intergroup contact and changes in styles of pictorial representations has not been fully established in an experimental setting. Some previous studies in experimental semiotics have suggested a relationship between group size and semantic transparency of pictorial representations (Fay *et al*. 2008; Rogers *et al.* 2013; for a review of experimental semiotic studies, see Galantucci *et al.* 2012), but have not investigated the role that intergroup contact might play, nor have they studied style as a factor distinct from transparency. Healey *et al*. (2007) tested the effect of having a shared interaction history on the styles of representation adopted by pairs of drawers, comparing same-group pairs having a previous shared interaction history with different-group pairs at their first interaction; however, they did not look at the effects of group contact over time.

Here we use laboratory microsocieties to experimentally investigate the effects of intergroup contact on two aspects of pictorial representation, namely style (figurative or abstract forms) and transparency of meaning (ease of interpretation for outsiders). Drawing on methods developed to study the cultural evolution of graphical communication (Caldwell and Smith 2012; Fay *et al.* 2010; Garrod *et al*. 2007; Healey *et al*. 2007; Tamariz and Kirby 2014), we use a Pictionary-style task in groups, where participants communicate given concepts to each other by drawing alone. The task was embedded in a design that simulated contexts of cultural exchange and isolation as experimental conditions by manipulating the degree of interaction between groups of participants. We then looked at the differences in transparency and style of the drawings resulting from the game sessions by running two surveys with naïve participants. We hypothesised that: (1) pictorial representations produced in contexts of group isolation are more difficult for outsiders to interpret than those produced in contexts of intergroup exchange; and (2) this difference in transparency of meaning is due to a difference in style of representation; specifically, the pictorial representations resulting from the contact condition will tend to be figurative (i.e. to contain inter-subjectively recognisable objects, living beings, scenes), whereas pictorial representations from the isolation condition will tend to be more abstract in character. The rationale is that, differently from isolated groups, in contexts of contact the need to communicate with members of different cultural groups causes pictorial signs to retain figurativeness and maintain accessibility to potentially any audience. Signs used in contexts of isolation are more free to develop symbolic, abstract and other features that reduce comprehensibility to outsiders.

We note that the distinction figurative–abstract does not entirely overlap with the Peircean distinction iconic–symbolic usually found in the experimental semiotics literature (e.g. Caldwell and Smith 2012; Garrod *et al.* 2007). This is because sometimes figurative pictorial signs are not iconic in the original Peircean sense (Peirce 1860, W2.56): a pictorial sign might bear visual resemblance to some recognisable things, yet it might not bear direct perceptual resemblance to its intended referent; for example, a clearly recognisable drawing of New York cityscape that is meant to stand for the meaning ‘jazz’ is a figurative sign but not a Peircean icon for ‘jazz’, as it does not bear direct perceptual resemblance to jazz music.

The distinction between figurative and abstract styles of representation is relevant to important developments in the study of human cultural evolution and the origin, significance and development of the earliest pictorial signs. In particular, by suggesting possible evolutionary paths for figurative and abstract signs, and their relation to the social contexts of production, this study might contribute to the debate on whether or not early geometric patterns produced by hominins served a symbolic function (Henshilwood *et al.* 2018; Hodgson 2014, 2016, 2019; Mellet *et al*. 2018; Tylén *et al*. 2017).

## Methods

The study is composed of two phases. In Phase 1 (Data production) laboratory microsocieties played a Pictionary-like task in one of three conditions: isolation, contact or a control condition, which were simulated by manipulating the degree and structure of interaction between participants. The drawings produced at this stage were then used as stimuli in two surveys run in Phase 2: in one, naïve participants were asked to match the drawings with their meanings; in the other, other naïve participants had to say whether the drawings contained recognisable figures or not. Ethical approval was granted by Durham University Anthropology Committee. All participants gave their consent.

### Phase 1 – data production

#### Participants

Fifty-four students from Durham University participated in exchange for a lottery prize of £50 in Amazon vouchers.

#### Apparatus

A5 notebooks and black felt-pens were used for drawing. Experimenters used stopwatches to time group performance, and a group sheet to collect playing times and any cheating episodes in order to later assign rewards to participants.

#### Stimuli

Two lists, A and B, each of 12 target words, were selected from two merged databases of English words (Brysbaert *et al.* 2014; Stadthagen-Gonzalez and Davis 2006) containing measures of psycholinguistic variables, such as concreteness (the degree to which a word refers to a perceptible entity, measured on a rating scale from 1, very abstract, to 5, very concrete) and imageability (how easily a word elicits a mental picture of its referent, measured on a rating scale from 1, low imageable, to 7, highly imageable, and converted into a scale from 100 to 700). Half of the words in each list were highly abstract (concreteness score ≤ 2) and half highly concrete (concreteness score ≥ 4), and all had low inter-subjective variability (SD ≤ 1.1). The words were chosen to be potentially confusable in their graphical representation (e.g. *fame* and *glory*; or *sweat* and *anxious*), and the degree of imageability was controlled (all words had medium imageability scores, i.e. 300–500). List A included *actor*, *blaze*, *gear*, *mill*, *sweat*, *trap* (concrete) and *anxious*, *envy*, *fame*, *gain*, *gloom*, *glory* (abstract); list B included *cloth*, *jean*, *midwife*, *nylon*, *patch*, *womb* (concrete) and *ancient*, *bliss*, *dodgy*, *smart*, *spooky*, *wise* (abstract).

#### Procedure and experimental design

A total of 54 participants took part in the experiment. They were split into six groups of nine. Two groups played in the isolation condition, two in the contact condition and two in a control condition controlling for effective population size. These conditions differed in how the groups were organised, as specified below. We ran each condition twice, once with each wordlist (A or B).

Participants were informed that they were about to play a drawing game similar to the game Pictionary: they had to communicate concepts only by drawing, with no speech, gestures, numbers, letters, mathematical or currency symbols. In each round of the game there was one director (who had to draw), one matcher (who had to guess) and either one or seven observers, depending on condition (see below). At all times, a public copy of the full list of possible answers (in alphabetical order) was always visible to the whole group.

At the beginning of each round, each director was given a randomised list of the 12 target words, and was required to draw them one by one in the given order; each drawing constituted one trial. This list was only visible to the director, and the random order of the list changed with each round.

**Figure 2.**
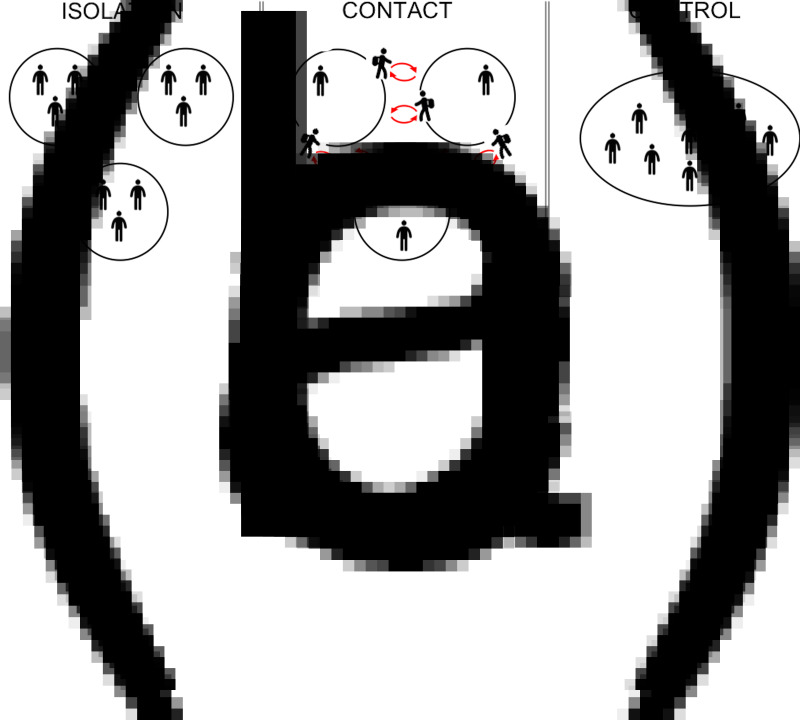
A schematic illustration of the three conditions. (a) Isolated groups – in each mini-group, each participant plays only with same-group members; (b) contact groups – in each mini-group, each participant alternates playing with same-group members and different group members; and (c) control group – one large group of nine people, each interacting in equal measure with each other.

On each trial, the director drew until the matcher said ‘stop’; the matcher then pointed at the answer on the public answer list and the director gave feedback – for correct answers, they put a tick next to the drawing, otherwise a cross. In case of wrong answer, directors were not allowed to then reveal the correct answer. Matchers only had one guess, after which the director moved onto the next word, regardless of whether the guess was correct or not. This process was repeated for 12 trials, i.e. until the full list of words had been completed. The participants then changed roles, in a way determined by experimental condition, as described below (see also Figure 2).

In all conditions, speed and accuracy were encouraged through a prize–penalty system. The playing time of each group in each round was recorded and assigned individually to each of the members. At the end of each game, each participant had a record of the overall time they spent playing. The fastest three participants in each session received a £20 coupon each, the second three a £15 coupon, the last three a £10 coupon. A 7 s penalty was added to group playing time for every incorrect guess, and 14 s for each cheating episode (i.e. talking and using numbers or letters).

##### Isolation condition

Here each group of nine participants was split into three mini-groups of three, and each participant only ever interacted with the two other members of their mini-group. After each round of 12 drawing trials, the three participants rotated roles. This meant that over six rounds each of the three different roles (director, matcher, observer) was counter-balanced. Such six-round blocks (henceforth, ‘home blocks’) were iterated six times in a row. This created a total of 36 rounds and 432 drawings (as did both other conditions, described below).

##### Contact condition

Here each group of nine participants was, as in the isolated condition, split into three mini-groups of three, but they also had occasional contact with members of other mini-groups. Mini-groups alternated a home block with a ‘travel block’, where each member of a mini-group interacted with the one member from each of the other two mini-groups. After completing a travel block, participants went back to their home mini-group to play another home block. Each mini-group alternated home blocks and travel blocks three times, for a total of six blocks.

##### Control condition

Here each group of nine participants was not split into mini-groups, and so each participant interacted with the eight other members of their group: instead of one director, one matcher and one observer (as per the other conditions), there was one director, one matcher and seven observers. Each participant interacted in equal measure with each of the others, and the total number of rounds was identical to the groups in the other conditions. This condition controls for effective population size, i.e. for the total number of individuals that come into contact with the evolving set of pictorial signs. This is necessary because otherwise the effective population size would be a confounding variable: while in the isolation condition the effective population size is 3, in the contact and control conditions it is 9.

In summary, the difference between conditions lies in the structure of interaction between participants (see Figure 2). A difference in drawing transparency and style between contact and isolation conditions is likely to be due to the presence/absence of intergroup contact and not to effective population size, if a similar difference is found between contact and control conditions. Also, participants played director and matcher roles, and played these roles with the same partners, at a lower rate in the control condition than the other two conditions. We account for this feature of the design when interpreting the results (see Discussion). Full details of the ordering and counter-balancing employed in each condition are provided as Supplementary Information.

**Figure 3.**
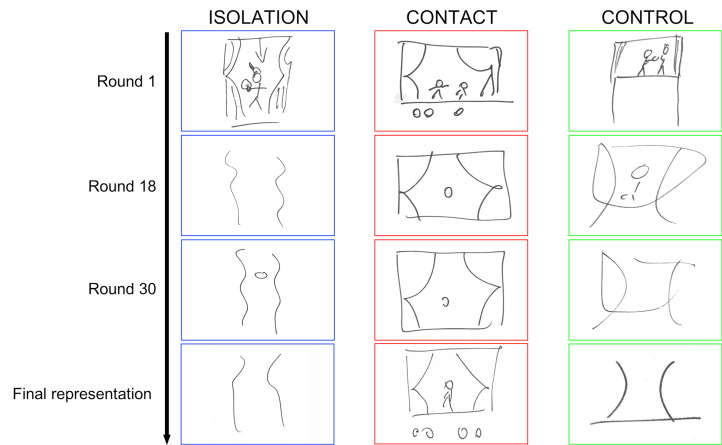
Drawings of ‘actor’ from successive rounds from each experimental condition (Phase 1). The final drawings (bottom row) were later used as stimuli in two surveys on transparency and style of representation (Phase 2).

In all conditions participants were asked, after completing the game, to privately draw all of the concepts individually on a set of cards labelled with the target words. They were instructed to draw them in the way that they would do it for their home groups. This was done in order to capture sign types, rather than tokens of types. Tokens can differ from their types, sometimes dramatically so, when they are produced in an episode of interaction under time pressure. It was these drawings that were used in the surveys in Phase 2. Figure 3 shows representative examples of these final drawings (bottom row), alongside drawings from previous rounds (the full set of drawing is available at Granito *et al.* 2018).

### Phase 2 – surveys

#### Transparency survey

In this survey, naïve participants (i.e. people who did not take part in Phase 1) were asked to match different drawings from the Pictionary game with their meanings.

##### Participants, stimuli and procedure

A total of 180 people were recruited through the online platform Prolific and took part in an online survey designed with Qualtrics in exchange for a payment of £6/hour. Stimulus materials were the 648 individual drawings produced at the end of Phase 1. Each participant was presented with the full list of 12 target words (i.e. all words from List A or List B, in alphabetical order). They were then presented with 36 drawings from the end of Phase 1, one at a time, and asked to guess which of the 12 possible target words the drawing represented. In each case, these 36 drawings were all sampled from the same condition (isolation, contact, or control) and the same list (A or B). In other words, each participant in this survey saw drawings only from one condition and one list, but which condition and which list varied between participants.

#### Style survey

In this survey, naïve participants (different to those from both Phase 1 and the Transparency Survey) were asked to say whether drawings contained inter-subjectively recognisable figures or not. This provides a measure of whether the drawings had a figurative or abstract style. (We mention here in passing that other equally reliable measures are possible that treat the abstract–figurative distinction as a continuum between two poles rather than a binary category, e.g. see Tamariz and Kirby 2014.) Note incidentally that style of representation (abstract or figurative) does not overlap with complexity (simple vs complex); abstract drawings might be very complex, e.g. intricate doodles with no intended reference.

##### Participants, stimuli and procedure

The whole dataset of 648 individual drawings produced at the end of Phase 1 was presented to each of 10 participants (students at Durham University), giving a total of 6480 style judgments. Order of presentation was randomised. Participants were shown a target drawing and were asked to indicate (with a ‘yes’ or ‘no’ on an answer sheet) whether, in their opinion, the drawing included things that the participant could clearly recognise and that some other reasonable person would also clearly recognise.

All survey data are available at Granito *et al*. (2018).

### Statistical information

To estimate the effect of the experimental conditions on the transparency of meaning of drawings and their style of representation, interpretation accuracy and style were analysed by drawing with aggregated binomial regression models using a logit link function; models were run with McElreath's Bayesian *rethinking* R package (McElreath 2016; R Core Team 2017). We constructed multilevel models and generated posterior estimates using *rstan* package's Hamiltonian Monte Carlo. The equivalent frequentist models are available in the Supplementary Information.

We constructed two models, ‘Transparency’ and ‘Style’, with a binary response variable for correct interpretation and figurativeness, respectively. The models included the following fixed variables, each with an associated coefficient (slope): *β*, condition (isolation, contact and control, recoded into dummy variables with isolation as ‘00’, i.e. the baseline, for ease of interpretation); kind of concept (abstract/concrete); and list of concepts (list A/list B). The models also included separate varying intercepts (with normally distributed hyperparameters to describe the standard deviation of the population of intercepts) for each drawing and for each concept represented. For Transparency only, we also specified a varying intercept for questionnaire, since – for practical necessities – in the transparency survey, drawings were sorted into different questionnaires taken by different sets of participants.

In order to assess the effect of condition, we compared each model for out-of-sample deviance (WAIC) against a null model, which only included the intercepts representing the multilevel structure and the two covariates kind of concept and list of concepts, but no condition coefficients (i.e. effectively, the isolation condition).

For relevant fixed variable coefficients, *β*, we quote the posterior mean, standard deviation and the highest posterior density interval (89% HPDI), in units of log-odds (negative and positive effects of the predictor variable on the response variable compared with the baseline category, isolation, lie either side of zero). To compare the absolute effect of each condition on the probability of the outcome, we extracted posterior samples of the models’ estimates for the condition parameters and converted them into probability distributions by applying the logistic function (McElreath 2016). See Supplementary Information for the statistical models.

## Results

### Quantitative results

#### Are drawings from the contact condition more likely to be interpreted correctly than drawings from the isolation and control condition?

**Figure 4.**
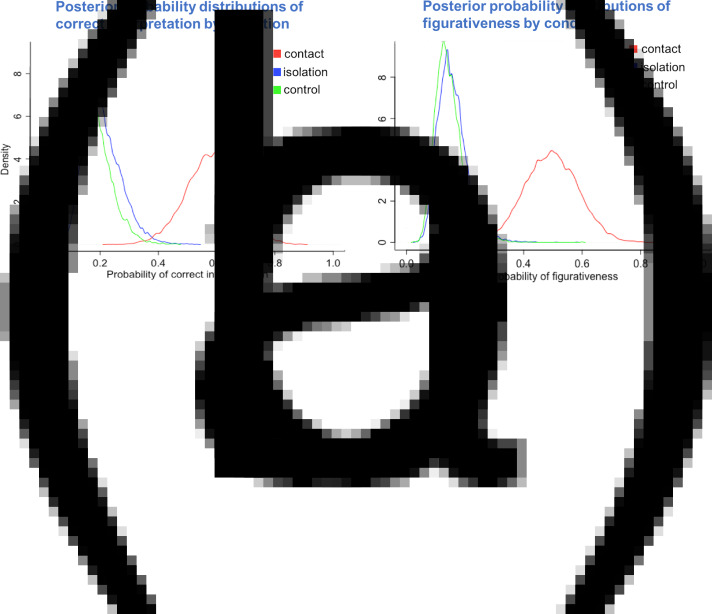
Posterior probability distributions from the (a) transparency and (b) style Bayesian models. Drawings from the contact condition were more likely to be correctly interpreted, and more likely to be judged as figurative (*n* = 648).

Yes. The Transparency model had a lower WAIC than the null model (WAIC_transparency_ = 6629.9, WAIC_null_ = 6634.5, with WAIC_transparency_ weighting 91%) and the standard error for the difference between the two WAIC scores was a little smaller than their difference (difference, dWAIC = 4.7, dSE = 3.28). This indicates that the condition parameters (in the Transparency model) may be a useful predictor of out-of-sample data; see Figure 4a.

There was a positive effect of contact against the baseline category isolation (*β*_contact_ mean = 1.75, SD = 0.20, HPDI = 1.44–2.07), whereas there was no clear effect of control over isolation in the log-odds of correct interpretation (*β*_control_ mean = −0.21, SD = 0.20, HPDI = −0.51 to 0.11). Comparing the median estimates from the posterior probability of correct interpretation between conditions, we found that the probability of correct interpretation for drawings from the contact condition was 38% higher than the isolation condition (HPDI = 27% to 47%) and 42% higher than the control condition (HPDI = 30–50%), whereas there was only a very small difference in probability between control and isolation (3% advantage of isolation over control, HPDI = 2–10%). Figure 4a illustrates the predicted effect of the conditions on the probability of correct interpretation and shows a trend that is consistent with our hypothesis: drawings coming from the contact condition were more likely to be interpreted correctly than drawings coming from the isolation or control conditions, which had instead similar low interpretation accuracy.

#### Are the drawings from the contact condition more likely to be figurative than the drawings from the isolation and control condition?

Yes. The Style model had a lower WAIC than the null model (WAIC_style_ = 5032.5, WAIC_null_ = 5043.0, with WAIC_style_ weighting 99%), but noting that the standard error for the difference between the two WAIC scores was a little smaller than their difference (difference, dWAIC = 10.5, dSE = 9.74). This provides some evidence that the condition parameters are a useful predictor of out-of-sample data; see Figure 4b.

There was a positive effect of contact against the baseline category isolation (*β*_contact_ mean = 1.71, SD = 0.20, HPDI = 1.41–2.03), whereas there was no clear effect of control over isolation in the log-odds of a drawing being figurative (*β*_control_ mean = −0.09, SD = 0.21, HPDI = −0.41–0.25). Comparing the median estimates for the posterior probability distributions between conditions, we found that the probability of figurativeness for drawings from the contact condition was 34% higher than the isolation condition (HPDI = 23–44%) and 35% higher than the control condition (HPDI = 23–46%), whereas there was only a 1% probability advantage of isolation over control (HPDI = 4% to 7%). Figure 4b illustrates the predicted effect of the conditions on the probability of figurativeness and shows a trend that is consistent with our hypothesis: drawings coming from the contact condition were more likely to be figurative than drawings coming from the isolation or control conditions, which had instead similar low probabilities of figurativeness.

A methodological issue of experiments where participants repeatedly interact in groups is that data (in our case, drawings) produced within a group are not independent. To address this issue, we ran two additional models (one for Transparency and one for Style) including a cluster variable for group as a random variable generating a varying intercept (McElreath 2016). We found that the new models imply nearly identical predictions to the original models and that the effect of condition was essentially the same (see Supplementary Information for details).

#### Qualitative results

In this section, we will briefly discuss the processes of change in the drawings during the game from a qualitative point of view, informed by the results of the quantitative analysis and by referring to the representative sample shown in Figure 3.

In the isolation condition, over repeated interactions with same-group members, representations change from figurative and detailed depictions of objects and people to extremely simplified lines and abstract shapes, so much that they lose any resemblance to the things of the world. The final pictorial representations typically need group-specific cultural information to be interpreted, therefore outsiders are less likely to interpret their meanings correctly. This process mirrors the findings of previous work in the evolution of graphical communication systems (Caldwell and Smith 2012; Garrod *et al*. 2007). However, unlike previous observations (Fay and Ellison 2013; Fay *et al*. 2008), the same processes of stylistic simplification and increase in opacity take place in both the isolation and the control condition, which differ for number of group members (three in isolation vs nine in control). This suggests that the difference in effective group size and rate of playing director/matcher roles did not affect the change in style and degree of transparency of drawings.

A different process of change is observed in the contact condition. During the initial home rounds, just as in the initial stages of the isolation and control conditions, participants develop a shared common ground within their home groups and start to establish initial group-specific conventions using increasingly stylised forms. However, in the first travel block, where participants have to interact with different-group members, those initial conventions do not allow effective communication. Therefore, participants switch to a figurative strategy in which the elements of the drawings ‘look like something’ and require less group-specific information to be interpreted. A similar return to a figurative strategy in pairs of participants which do not share an interaction history was also observed in Healey *et al*. (2007). However, in our case, shared interaction history does not appear to play a role in producing the final effect. During the game, participants repeatedly alternate travel blocks and home blocks; when participants go back to their home groups after a travel block, in the early stages of the game they just tend to switch back to their home stylised conventions; however, as the game progresses and the encounters with different-group members iterate, participants tend to adopt the figurative strategies developed during the travel blocks even when playing with same-group members, with which they do share an interaction history. This is probably because storing and using a single version of a representation to use in any occasion of interaction is less cognitively heavy than storing multiple representations, one for each occasion of interaction. Playing under time pressure, participants presumably selected for each meaning the graphical representation they found to be most effective in communicating quickly. Over time, drawings in the contact condition may become slightly less detailed so as to reduce the drawing effort, but they still maintain largely inter-subjectively recognisable figures.

## Discussion

Overall, these findings support the hypothesis that intergroup contact influences the development of styles and transparency of pictorial representation. Our results show that drawings from the contact condition are more transparent and more figurative than drawings from the isolation and control conditions. In other words, compared with the contact condition, drawings evolve to become abstract and opaque in the two conditions where there was no intergroup contact. This appears to be unaffected by whether the no-contact effective group size was the same (control) or smaller (isolation) than the group size in the contact condition. We conclude that the effect is due to the possibility for participants in the contact condition of having to communicate with outsiders: as a consequence of the need to make representations accessible to potentially any audience, style retains figurativeness and the drawings retain external interpretability.

In our experimental design, two unavoidable confounds are theoretically possible but empirically implausible (for a similar case, see Garrod and Doherty 1994). The first is that experience of playing with the same individual was lower in the control condition than in the other two. This is a direct consequence of keeping task experience and total trial numbers balanced across conditions. The second is that there was a lower active participation rate (i.e. the frequency of engagement of a participant as either director or matcher rather than observer) in the control condition than in the other two. This is a direct consequence of creating one large group of nine individuals but keeping constant the total number of trials. However, it seems extremely unlikely that low same-partner interaction rate and low active participation rate in the control condition would encourage the evolution of abstract pictorial signs. If anything, fewer interactions produce less abstract signs (Garrod *et al*. 2007), and we would expect active engagement to generate more abstract graphics as a result of shared attention and learning. As such, it is more plausible that the control condition exhibited a similar evolution of abstract drawings to that of the isolation condition because of the absence of intergroup contact rather than for a lower same-partner interaction rate or a lower active participation rate.

**Figure 5.**
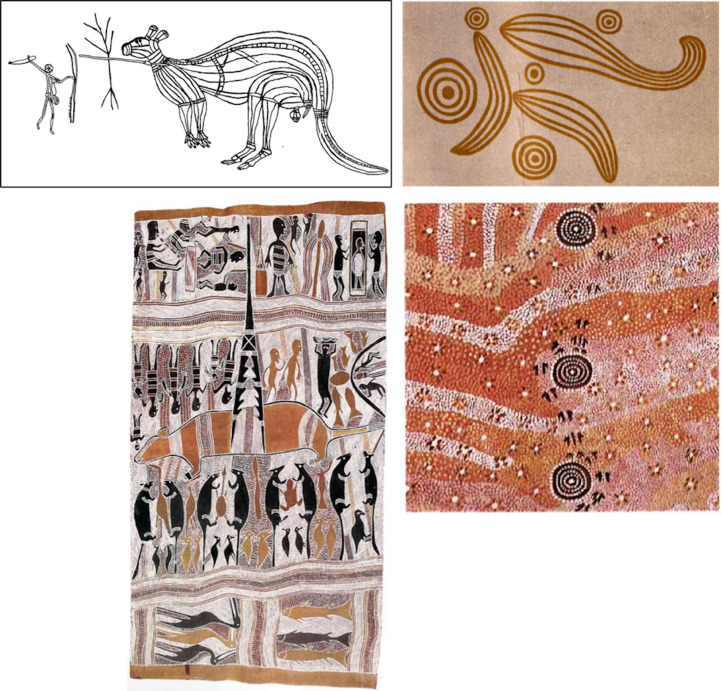
Aboriginal art as a real-world example. Left: examples of Arnhem Land rock art (top, from Lewis 1988) and bark painting (bottom, Narritjim Maymuru, Bamabama 1976), mainly presenting figurative motifs. Right: examples of Western Plateau rock art (top, from Basedow 1903) and painting (bottom, Charlie Eagle Tjapaltjari, Wallaby Dreaming in the Sandhills 1977), mainly presenting abstract motifs.

As a possible real-world example of this effect in action, consider two different areas of Aboriginal Australia, Arnhem Land and the Western Plateau, and compare their artistic productions. At the time of European contact, Arnhem Land was populated by a large number of high-contact Aboriginal groups engaged in intense networks of ceremonial and commercial exchanges (Davidson 1935; Grey, 1841; Mulvaney 1976; Petri 1950), whereas groups in the Western Plateau were fewer and more isolated, entertaining only rare or very sporadic interactions (Birdsell 1976; Mulvaney 1976). In both contexts, visual art played an important role in religious gatherings and covered a storytelling function by encoding ancestral myths and events from everyday life in conventionalised visual forms (Layton 1992a). However, the visual forms adopted to illustrate those stories differed greatly between the two areas. In the Arnhem Land groups, there was a strong prevalence of silhouette traditions including recognisable animal and human shapes (Layton 1992a, see figure 5 left, top and bottom). By contrast, in Western Plateau groups, artworks prevalently included highly stylised, geometric motifs, such as concentric circles, semicircles, wavy lines (Morphy 1998; see Figure 5 right, top and bottom). Western Plateau motifs were also difficult to interpret for ethnographers, and in the lack of local informants, the meanings of many motifs remained obscure (e.g. see Basedow 1903; Mountford 1937, 1955). This difference in forms of representation between the two areas occurred across material supports and pictorial means, for example it can be found in rock art motifs (Layton 1992a; Taylor 2005; Figure 5, top, left and right) as well as in portable paintings (Morphy 1998; Figure 5, bottom, left and right). The silhouette–geometric distinction is widely overlapping with our distinction between figurative and abstract styles. This analysis is of course speculative, but it nevertheless illustrates how the effects observed in our study might translate into real-world phenomena. (A quantitative study on this case is currently in preparation.)

More generally, and regardless of whether the above speculation is correct, our results contribute to the ethnographic literature by providing an experimental demonstration that correlated changes between intergroup contact on one side and representational transparency and figurativeness on the other are likely to be causal. In our experiment, the increase in figurativeness and transparency occurs exactly and only when the need for communication with outsiders is present. Of course, intergroup contact sometimes occurs between groups that have no intention to communicate with each other, and in these cases we should not expect that phenomenon to occur.

At the same time, the experimental context is clearly idealised, and real-world scenarios are likely to present more noise. Artistic representation in particular is likely to be influenced by other factors related to political context, religious ideas, identity marking and ostentatious displays of skill or creativity, which might drive greater elaboration and improvisation in design by individuals seeking to ‘stand out’ from the crowd. In this paper, we are isolating the specific factor of intergroup communication and simulating one particular process. An important goal for future research is to systematically analyse style in real-world pictorial representations, in particular considering artistic representations with a storytelling function, with the goal to determine the relative strength and generality of the link between intergroup contact and representational style.

Another parallel example in the real world is language change. Research in sociolinguistics and language evolution has shown the existence of a correlation between the degree of contact of a community of speakers (among other socio-demographic factors) and language complexity (Lupyan and Dale 2010; Reali *et al.* 2018). Languages spoken in societies of strangers (high-contact, large sized, loosely-knit communities with small amounts of socially shared information) are more lexically and morphologically transparent and regular, and less redundant than languages spoken in societies of intimates (low-contact, small sized, tightly knit communities with large amounts of socially shared information; Trudgill 2011). This is generally thought to be due to the large-scale learning by non-native adults taking place in societies of strangers, which would act as a selective filter for complexification (an example of this is the process of pidginisation; McWhorter 2011; Wray and Grace 2007). In other words, in high-contact communities, languages become easier for non-natives to understand and learn, whereas in small isolated communities, languages are more difficult for non-natives to understand and learn. Our study shows that this correlation, between degree of contact of a community of speakers on the one hand, and transparency of meaning on the other, might be causal, for reasons that are in line with sociolinguistic theory. It may be the case that intergroup contact is a driver of communicative transparency regardless of the specific communication medium.

## Supplementary material

For supplementary material accompanying this paper visit http://dx.doi.org/10.1017/ehs.2019.8. click here to view supplementary material

## Research transparency and reproducibility

Dataset of drawings and survey data are deposited at Figshare: https://figshare.com/s/a4f03733f97a2ddf661d
