# Supplementary material for: Style of pictorial representation is shaped by intergroup contact
Source: Evol Hum Sci. 2019 Jul 23;1:e8. doi: 10.1017/ehs.2019.8 (PMC10427304; doi:10.1017/ehs.2019.8)
Supplement: Supplementary file 1 [file S2513843X19000082sup001.docx]

Supplementary Information

[Supplementary Methods 2](#_Toc514936548)

[Supplementary Results 3](#_Toc514936549)

[Supplementary Tables 6](#_Toc514936550)

# Supplementary Methods

## A note on stimuli

The lists of stimuli words used in the drawing game did not include any distractor. Distractors were not needed because our procedure made guessing the last few concepts on the list not trivial for participants. This was for a combination of reasons: 1) when a wrong answer was given, the right answer was not revealed, which made it impossible to exclude words from the set of potential answers to the following trials. 2) Even in later rounds of the game, when accuracy would improve, it was still extremely unlikely for participants to proceed by elimination considering that each participant had to deal with the whole list of 12 words in each round and that the average memory span for individual words is about 5 or 6 (Baddeley, 2000). Participants would have needed to remember the last 9 or 10 words to have some chances to guess the last two. 3) This was made even harder by the fact that the words were confusable and that confusable words came sometimes in triplets or sets of 4.

## Bayesian models: structure, priors and chain convergence

1. **Transparency model**

The code of the Transparency model is the following:

m1 <- map2stan(

      alist(

        Correct ~ dbinom (Total_trials, p),

        logit(p) <- a +

        + a_Concept[Concept]

        + a_Questionnaire[Questionnaire]

        + a_Drawing[Drawing]

        + b_List*List

        + b_KindConcept*Kind_of_Concept

        + b_Contact*Contact

        + b_Control*Control,

        a ~ dnorm(0,10),

        a_Concept[Concept] ~ dnorm(0, sigma_concept),

        a_Questionnaire[Questionnaire] ~ dnorm(0, sigma_questionnaire),

        a_Drawing[Drawing] ~ dnorm(0, sigma_drawing),

        b_List ~ dnorm(0,10),

        b_KindConcept ~ dnorm(0,10),

        b_Contact ~ dnorm(0,10),

        b_Control ~ dnorm(0,10),

        sigma_concept ~ dcauchy(0,1),

        sigma_drawing ~ dcauchy(0,1),

        sigma_questionnaire ~ dcauchy(0,1)

        ),

      data = surveydata, warmup = 1000, iter = 4000, chains = 3

      )

Concept, Questionnaire and Drawing are included as random variables generating varying intercepts (varying intercepts priors). List (A, B) and Kind of Concept (abstract, concrete) are covariates which are included as fixed variables. The fixed factor Condition (Isolation, Contact, Control) has been recoded into dummy variables, where Isolation is the baseline: Isolation (0,0), Contact (1,0), Control (0,1).

The parameters for contact, control, kind of concept, list of concepts, and the mean intercept were assigned normal distributions (mean=0, SD=10). The varying intercept parameters for drawing, concept, and questionnaire were assigned normal distributions with mean at 0 and SD as a hyperparameter, sigma, which takes a half-Cauchy distribution (0, 1) (McElreath, 2016).

We ran 3 Markov chains of 4000 iterations (with 1000 warmup), all of which converged (R^=1).

The model gave this output:

                     Mean StdDev lower 0.89 upper 0.89 n_eff Rhat

a                   -1.36   0.41      -2.01      -0.70  1620    1

b_List              -0.12   0.45      -0.82       0.60  1933    1

b_KindConcept        0.46   0.44      -0.22       1.17  1737    1

b_Contact            1.75   0.20       1.44       2.07  2896    1

b_Control           -0.21   0.20      -0.53       0.11  2863    1

sigma_concept        1.00   0.18       0.74       1.26  9000    1

sigma_drawing        1.06   0.06       0.96       1.14  2031    1

sigma_questionnaire  0.24   0.10       0.08       0.38   969    1

1. **Style model**

The code of the Style model is the following:

m1 <- map2stan(

  alist(

    Figurativeness ~ dbinom (TotalTrials, p),

    logit(p) <- a +

      + a_Concept[Concept]

    + a_Drawing[Drawing]

    + b_List*List

    + b_KindConcept*KindOfConcept

    + b_Contact*Contact

    + b_Control*Control,

    a ~ dnorm(0,10),

    a_Concept[Concept] ~ dnorm(0, sigma_concept),

    a_Drawing[Drawing] ~ dnorm(0, sigma_drawing),

    b_List ~ dnorm(0,10),

    b_KindConcept ~ dnorm(0,10),

    b_Contact ~ dnorm(0,10),

    b_Control ~ dnorm(0,10),

    sigma_concept ~ dcauchy(0,1),

    sigma_drawing ~ dcauchy(0,1)

  ),

  data = styleratings, warmup = 1000, iter = 4000, chains = 3

)

Concept, Questionnaire and Drawing are included as random variables generating varying intercepts (varying intercepts priors). List (A, B) and Kind of Concept (abstract, concrete) are covariates which are included as fixed variables. The fixed factor Condition (Isolation, Contact, Control) has been recoded into dummy variables, where Isolation is the baseline: Isolation (0,0), Contact (1,0), Control (0,1).

The parameters for contact, control, kind of concept, list of concepts, and the mean intercept were assigned normal distributions (mean=0, SD=10). The varying intercept parameters for drawing, concept, and questionnaire were assigned normal distributions with mean at 0 and SD as a hyperparameter, sigma, which takes a half-Cauchy distribution (0, 1) (McElreath, 2016).

We ran 3 Markov chains of 4000 iterations (with 1000 warmup), all of which converged (R^=1).

The model gave this output:

               Mean StdDev lower 0.89 upper 0.89 n_eff Rhat

a             -1.73   0.38      -2.32      -1.11  2755    1

b_List        -0.34   0.41      -0.99       0.30  3181    1

b_KindConcept -1.04   0.42      -1.73      -0.39  2941    1

b_Contact      1.71   0.20       1.41       2.03  3457    1

b_Control     -0.09   0.21      -0.41       0.25  3969    1

sigma_concept  0.92   0.19       0.62       1.18  5313    1

sigma_drawing  1.69   0.09       1.55       1.83  2772    1

# Supplementary Results

## Additional models

Drawings were produced in populations of participants who repeatedly interacted with each other: 6 populations in the isolation condition (the 6 isolated mini-groups), 2 populations in the contact condition (the interacting mini-groups in the 2 iterations of the contact condition), and 2 in the control condition (the large groups in the 2 iterations of the control condition). Drawings produced within the same population are not independent. We addressed this by running two additional models including the cluster variable “population” as a random variable generating a varying intercept (McElreath, 2016: 355-ff). In the new models, the effect of condition was essentially the same as in the original models.

### Additional Transparency model

The additional Transparency model (here named New) was:

New <- map2stan(

alist(

Correct ~ dbinom (Total, p),

logit(p) <- a +

a_Concept[Concept] +

a_Questionnaire[Questionnaire] +

a_Drawing[Drawing] +

a_Population[Population] +

b_List*List +

b_KindConcept*KindConcept +

b_Contact*Contact +

b_Control*Control,

a ~ dnorm(0,10),

a_Concept[Concept] ~ dnorm(0, sigma_concept),

a_Questionnaire[Questionnaire] ~ dnorm(0, sigma_questionnaire),

a_Drawing[Drawing] ~ dnorm(0, sigma_drawing),

a_Population[Population] ~ dnorm(0, sigma_population),

b_List ~ dnorm(0,10),

b_KindConcept ~ dnorm(0,10),

b_Contact ~ dnorm(0,10),

b_Control ~ dnorm(0,10),

sigma_concept ~ dcauchy(0,1),

sigma_drawing ~ dcauchy(0,1),

sigma_questionnaire ~ dcauchy(0,1),

sigma_population ~ dcauchy(0,1)

),

data = surveydata, warmup = 1000, iter = 4000, chains = 3

)

The model gave the following output (note that the 600+ parameters for Drawing were not displayed here for ease of reading):

Mean StdDev lower 0.89 upper 0.89 n_eff Rhat

a -1.44 0.46 -2.14 -0.71 1117 1.00

a_Concept[1] -0.93 0.43 -1.64 -0.28 1473 1.00

a_Concept[2] 0.31 0.44 -0.39 1.00 1186 1.00

a_Concept[3] 0.98 0.43 0.27 1.61 1422 1.00

a_Concept[4] -0.07 0.43 -0.78 0.61 1353 1.00

a_Concept[5] 0.87 0.43 0.18 1.53 1323 1.00

a_Concept[6] -0.13 0.44 -0.82 0.57 1329 1.00

a_Concept[7] 0.09 0.43 -0.55 0.81 1518 1.00

a_Concept[8] 0.37 0.43 -0.28 1.10 1309 1.00

a_Concept[9] -0.53 0.44 -1.21 0.17 1557 1.00

a_Concept[10] -0.06 0.43 -0.75 0.62 1341 1.00

a_Concept[11] -0.03 0.43 -0.70 0.67 1426 1.00

a_Concept[12] -0.76 0.44 -1.46 -0.06 1335 1.00

a_Concept[13] -1.36 0.44 -2.08 -0.68 1492 1.00

a_Concept[14] 1.04 0.43 0.40 1.77 1545 1.00

a_Concept[15] -0.30 0.43 -0.99 0.38 1337 1.00

a_Concept[16] -1.33 0.45 -2.01 -0.60 1754 1.00

a_Concept[17] 1.47 0.43 0.79 2.16 1407 1.00

a_Concept[18] 0.08 0.43 -0.60 0.75 1442 1.00

a_Concept[19] -1.57 0.45 -2.32 -0.88 1474 1.00

a_Concept[20] -0.22 0.43 -0.89 0.45 1532 1.00

a_Concept[21] 1.16 0.43 0.44 1.80 1394 1.00

a_Concept[22] 1.64 0.43 0.97 2.33 1565 1.00

a_Concept[23] 0.25 0.42 -0.38 0.95 1405 1.00

a_Concept[24] -0.95 0.45 -1.67 -0.25 1535 1.00

a_Questionnaire[1] 0.04 0.15 -0.19 0.29 1974 1.00

a_Questionnaire[2] -0.06 0.16 -0.31 0.18 1563 1.00

a_Questionnaire[3] 0.08 0.16 -0.14 0.36 1426 1.00

a_Questionnaire[4] -0.04 0.15 -0.27 0.20 2674 1.00

a_Questionnaire[5] -0.02 0.15 -0.27 0.21 3182 1.00

a_Questionnaire[6] 0.01 0.15 -0.24 0.23 3297 1.00

a_Questionnaire[7] -0.03 0.15 -0.27 0.23 2745 1.00

a_Questionnaire[8] -0.03 0.15 -0.25 0.22 2508 1.00

a_Questionnaire[9] -0.03 0.15 -0.28 0.20 3098 1.00

a_Questionnaire[10] -0.01 0.15 -0.25 0.22 3207 1.00

a_Questionnaire[11] 0.13 0.18 -0.10 0.42 919 1.00

a_Questionnaire[12] -0.03 0.15 -0.28 0.20 3414 1.00

a_Questionnaire[13] 0.09 0.16 -0.15 0.35 1982 1.00

a_Questionnaire[14] -0.11 0.17 -0.36 0.13 1227 1.00

a_Questionnaire[15] 0.05 0.15 -0.17 0.31 3112 1.00

a_Questionnaire[16] 0.12 0.17 -0.11 0.40 1052 1.00

a_Questionnaire[17] -0.14 0.18 -0.42 0.10 1058 1.00

a_Questionnaire[18] -0.01 0.15 -0.24 0.23 3587 1.00

a_Population[1] 0.19 0.35 -0.36 0.72 2063 1.00

a_Population[2] -0.19 0.36 -0.76 0.35 2226 1.00

a_Population[3] 0.12 0.36 -0.44 0.65 2838 1.00

a_Population[4] -0.11 0.35 -0.62 0.43 3144 1.00

a_Population[5] -0.13 0.28 -0.54 0.31 2387 1.00

a_Population[6] 0.20 0.29 -0.20 0.68 2298 1.00

a_Population[7] -0.41 0.30 -0.88 0.05 2143 1.00

a_Population[8] 0.06 0.27 -0.36 0.49 2512 1.00

a_Population[9] -0.07 0.27 -0.49 0.37 2763 1.00

a_Population[10] 0.35 0.29 -0.08 0.80 1625 1.00

b_List 0.03 0.52 -0.77 0.88 1303 1.00

b_KindConcept 0.45 0.44 -0.28 1.10 942 1.00

b_Contact 1.76 0.40 1.14 2.34 1674 1.00

b_Control -0.20 0.39 -0.78 0.42 2022 1.00

sigma_concept 1.00 0.18 0.72 1.25 3480 1.00

sigma_drawing 1.05 0.06 0.96 1.14 1717 1.00

sigma_questionnaire 0.16 0.10 0.00 0.28 347 1.01

sigma_population 0.40 0.19 0.12 0.67 1313 1.00

Figure 1. Posterior means and 89% highest density intervals for the additional transparency model


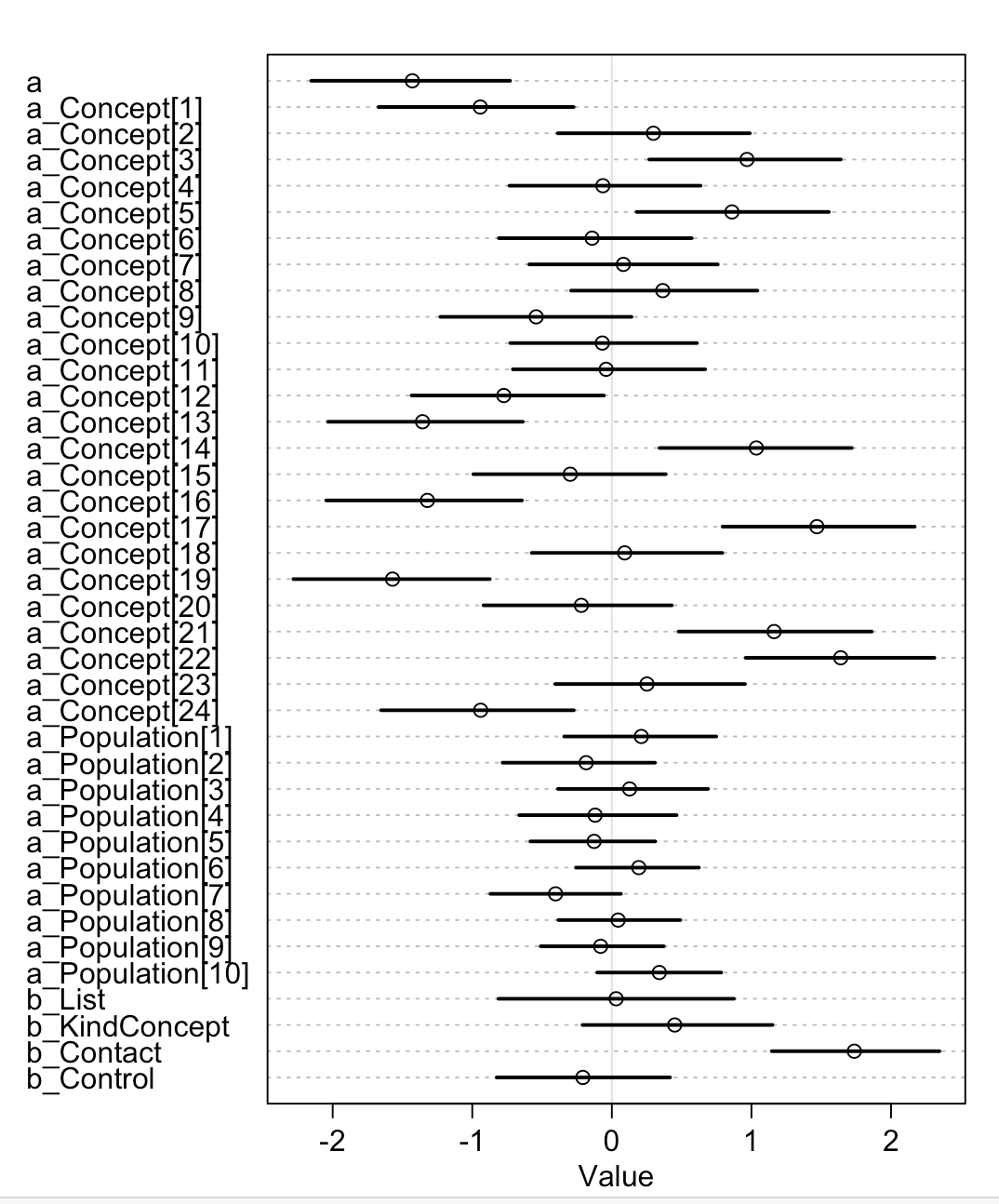


After accounting for the variance due to “population”, in the new model the effect of the condition was essentially the same as in the original model, only with a larger variance (see Table 1 and Figure 2). Similarly to the original model, in the new model there is a positive effect of contact against the baseline category isolation (β_contact_ mean = 1.76, SD = 0.40, HPDI = 1.14 to 2.34), whereas there was no clear effect of control over isolation in the log-odds of correct interpretation (β_control_ mean = - 0.20, SD = 0.39, HPDI = -0.78 to 0.42). Comparing the median estimates for the posterior probability distributions between conditions, we find that the probability of correct interpretation for drawings from the contact condition is 38% higher than the isolation condition (HPDI = 18% to 54%) and 40% higher than the control condition (HPDI = 20% to 59%), whereas there was a very small difference in probability between control and isolation (-3% in the control condition, HPDI = -14% to 1%). Figure 2 (left) illustrates the predicted effect of the conditions on the probability of correct interpretation according to the new model, and confirms the trend that was already shown in the original model (Figure 2 right), which is consistent with our hypothesis: drawings coming from the contact condition were more likely to be interpreted correctly than drawings coming from the isolation or control conditions, which had instead similar low interpretation accuracy.

Table 1 – Comparison of estimates between the new model and the old model

New model

Mean StdDev lower 0.89 upper 0.89 n_eff Rhat

a -1.44 0.46 -2.14 -0.71 1117 1.00

b_List 0.03 0.52 -0.77 0.88 1303 1.00

b_KindConcept 0.45 0.44 -0.28 1.10 942 1.00

b_Contact 1.76 0.40 1.14 2.34 1674 1.00

b_Control -0.20 0.39 -0.78 0.42 2022 1.00

sigma_concept 1.00 0.18 0.72 1.25 3480 1.00

sigma_drawing 1.05 0.06 0.96 1.14 1717 1.00

sigma_questionnaire 0.16 0.10 0.00 0.28 347 1.01

sigma_population 0.40 0.19 0.12 0.67 1313 1.00

Old Model

Mean StdDev lower 0.89 upper 0.89 n_eff Rhat

a -1.37 0.39 -1.97 -0.71 1319 1

b_List -0.11 0.43 -0.80 0.58 1586 1

b_KindConcept 0.46 0.43 -0.25 1.12 1318 1

b_Contact 1.76 0.20 1.45 2.07 2779 1

b_Control -0.20 0.20 -0.51 0.11 2832 1

sigma_concept 1.00 0.17 0.73 1.25 9000 1

sigma_drawing 1.06 0.06 0.97 1.15 1897 1

sigma_questionnaire 0.23 0.10 0.06 0.39 611 1

Figure 2. Posterior probability distributions from the new model (left) and old model (right)


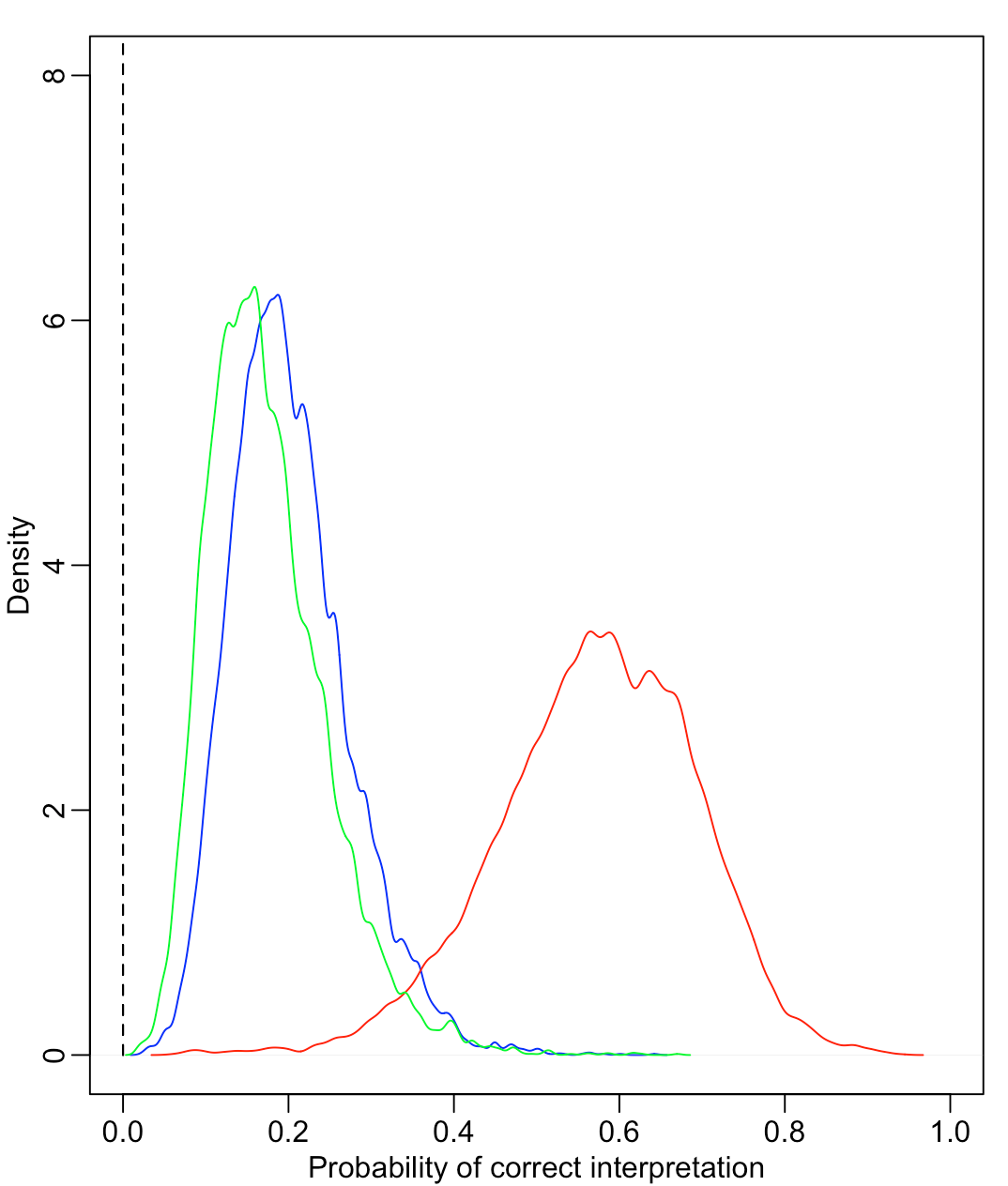

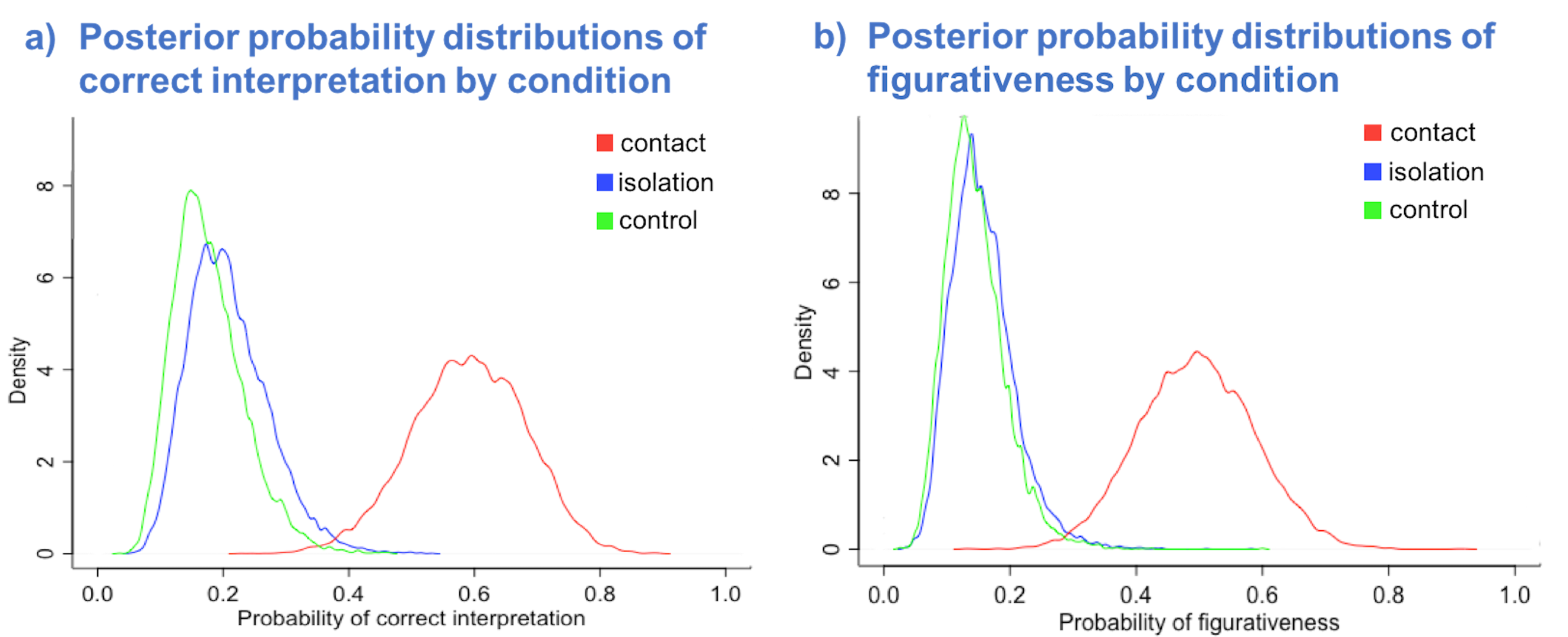


### Additional Style model

The additional Style model (here named NewStyle) was:

NewStyle <- map2stan(

alist(

ProportionFigurative ~ dbinom (TotalTrials, p),

logit(p) <- a +

+ a_Concept[Concept]

+ a_Drawing[Drawing]

+ a_Population[Population]

+ b_List*List

+ b_KindConcept*KindOfConcept

+ b_Contact*Contact

+ b_Control*Control,

a ~ dnorm(0,10),

a_Concept[Concept] ~ dnorm(0, sigma_concept),

a_Drawing[Drawing] ~ dnorm(0, sigma_drawing),

a_Population[Population] ~ dnorm(0, sigma_population),

b_List ~ dnorm(0,10),

b_KindConcept ~ dnorm(0,10),

b_Contact ~ dnorm(0,10),

b_Control ~ dnorm(0,10),

sigma_concept ~ dcauchy(0,1),

sigma_drawing ~ dcauchy(0,1),

sigma_population ~ dcauchy(0,1)

),

data = surveydata, warmup = 1000, iter = 4000, chains = 3

)

The model gave this output (note that the 600+ parameters for Drawing were not displayed here for ease of reading):

Mean StdDev lower 0.89 upper 0.89 n_eff Rhat

a -1.73 0.63 -2.70 -0.74 2800 1

a_Concept[1] 0.53 0.46 -0.21 1.26 5351 1

a_Concept[2] 0.30 0.46 -0.40 1.04 4407 1

a_Concept[3] -0.16 0.47 -0.88 0.60 5337 1

a_Concept[4] 0.87 0.47 0.08 1.58 4807 1

a_Concept[5] -1.42 0.53 -2.24 -0.58 6222 1

a_Concept[6] -0.77 0.48 -1.53 -0.03 5144 1

a_Concept[7] -0.23 0.48 -0.99 0.54 5218 1

a_Concept[8] -0.49 0.46 -1.26 0.22 4171 1

a_Concept[9] 0.64 0.47 -0.08 1.40 4667 1

a_Concept[10] -0.19 0.46 -0.92 0.56 4815 1

a_Concept[11] 0.85 0.47 0.12 1.61 4977 1

a_Concept[12] 0.06 0.47 -0.64 0.83 4858 1

a_Concept[13] 0.96 0.46 0.28 1.75 4662 1

a_Concept[14] 0.15 0.47 -0.56 0.93 4577 1

a_Concept[15] -0.81 0.49 -1.62 -0.05 5828 1

a_Concept[16] 0.75 0.46 0.01 1.49 4241 1

a_Concept[17] 1.41 0.47 0.67 2.14 4336 1

a_Concept[18] -0.26 0.46 -1.03 0.44 4452 1

a_Concept[19] 0.71 0.47 -0.04 1.44 4655 1

a_Concept[20] 0.27 0.45 -0.45 0.96 4561 1

a_Concept[21] -1.76 0.57 -2.63 -0.84 6501 1

a_Concept[22] -0.48 0.47 -1.20 0.28 4648 1

a_Concept[23] -0.80 0.51 -1.59 0.02 6000 1

a_Concept[24] -0.16 0.46 -0.90 0.58 4218 1

a_Population[1] 0.14 0.74 -1.01 1.30 6265 1

a_Population[2] -0.11 0.74 -1.19 1.13 5694 1

a_Population[3] -0.40 0.76 -1.59 0.74 5916 1

a_Population[4] 0.39 0.77 -0.74 1.65 5102 1

a_Population[5] 0.72 0.57 -0.18 1.60 3409 1

a_Population[6] 0.50 0.58 -0.37 1.42 3133 1

a_Population[7] -1.03 0.60 -2.01 -0.16 3188 1

a_Population[8] -0.49 0.57 -1.38 0.40 4573 1

a_Population[9] -0.58 0.58 -1.52 0.28 4520 1

a_Population[10] 0.83 0.58 -0.05 1.75 4171 1

b_List -0.45 0.74 -1.61 0.69 3296 1

b_KindConcept -1.02 0.42 -1.70 -0.36 3696 1

b_Contact 1.74 0.80 0.47 2.97 5072 1

b_Control -0.03 0.82 -1.33 1.20 4091 1

sigma_concept 0.92 0.18 0.64 1.20 5832 1

sigma_drawing 1.61 0.08 1.47 1.74 3020 1

sigma_population 0.91 0.34 0.42 1.36 2663 1

**Figure 3. Posterior means and 89% highest density intervals for the additional style model**
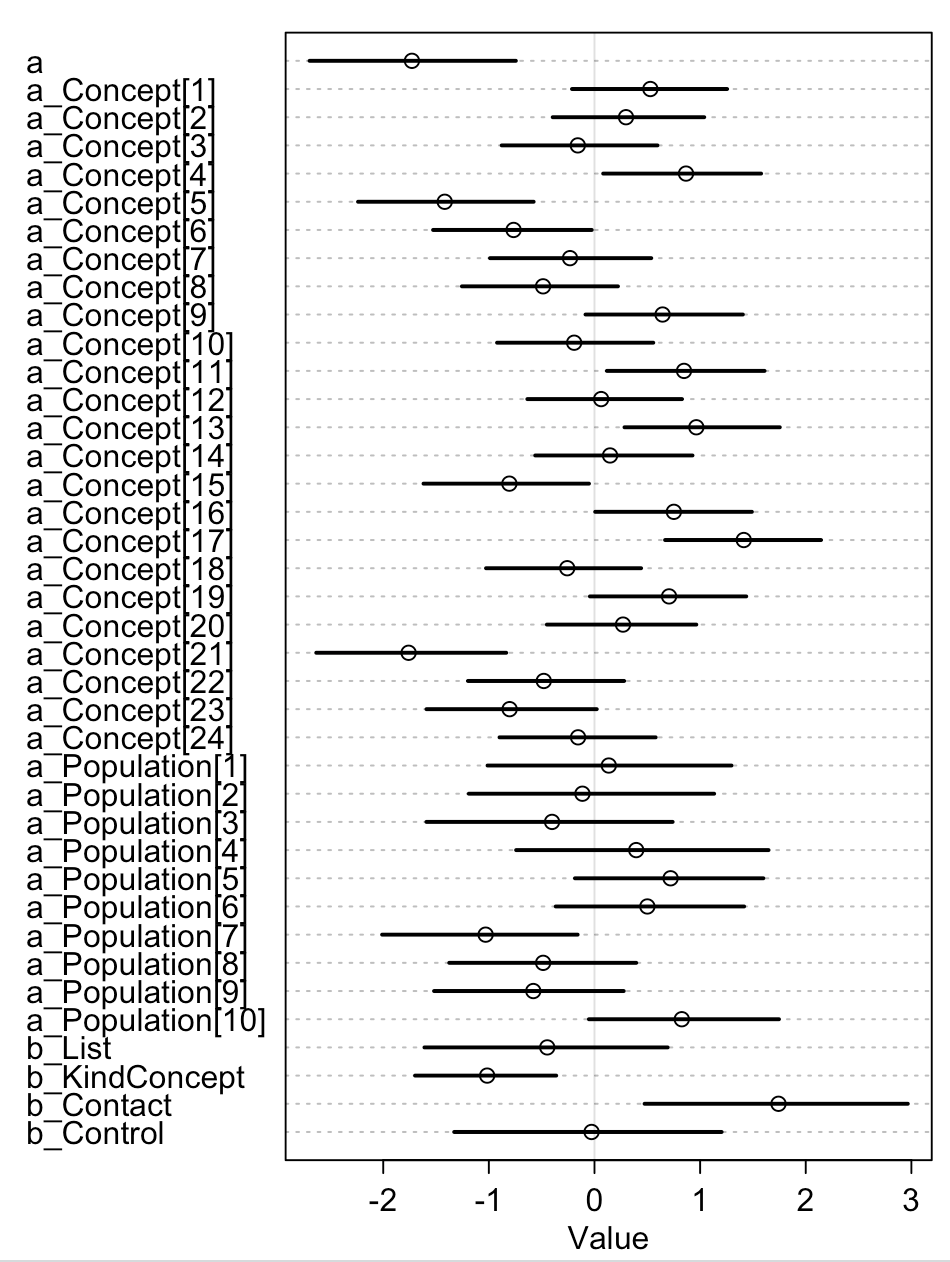


After accounting for the variance due to population, in the new model the effect of the condition was essentially the same as in the original model, but with a larger variance (see Table 3 and Figure 4). Similarly to the original model, in the new model there is a positive effect of contact against the baseline category isolation (β_contact_ mean = 1.74, SD = 0.80, HPDI = 0.47 to 2.97), whereas there was no clear effect of control over isolation in the log-odds of a drawing being figurative (β_control_ mean = -0.03, SD = 0.82, HPDI = -1.33 to 1.20). Comparing the median estimates for the posterior probability distributions between conditions, we find that the probability of figurativeness for drawings from the contact condition is 34% higher than the isolation condition (HPDI = 1% to 65%) and 33% higher than the control condition (HPDI = 4% to 67%), whereas there is essentially no difference in probability between control and isolation (-0.2% in the control condition, HPDI = -21% to 29%). Figure 4 (left) illustrates the predicted effect of the conditions on the probability of figurativeness according to the new model, and confirms the trend that was already shown in the original model (Figure 4 right), which is consistent with our hypothesis: drawings coming from the contact condition were more likely to be figurative than drawings coming from the isolation or control conditions, which had instead similar low probabilities of figurativeness.

Table 3 – Comparison of estimates between the old and new model

New model

Mean StdDev lower 0.89 upper 0.89 n_eff Rhat

a -1.73 0.63 -2.70 -0.74 2800 1

b_List -0.45 0.74 -1.61 0.69 3296 1

b_KindConcept -1.02 0.42 -1.70 -0.36 3696 1

b_Contact 1.74 0.80 0.47 2.97 5072 1

b_Control -0.03 0.82 -1.33 1.20 4091 1

sigma_concept 0.92 0.18 0.64 1.20 5832 1

sigma_drawing 1.61 0.08 1.47 1.74 3020 1

sigma_population 0.91 0.34 0.42 1.36 2663 1

Old model

Mean StdDev lower 0.89 upper 0.89 n_eff Rhat

a -1.74 0.39 -2.35 -1.13 2479 1

b_List -0.35 0.41 -0.99 0.31 3109 1

b_KindConcept -1.03 0.43 -1.71 -0.37 2881 1

b_Contact 1.71 0.20 1.38 2.02 3486 1

b_Control -0.10 0.21 -0.43 0.23 4079 1

sigma_concept 0.93 0.18 0.63 1.19 5143 1

sigma_drawing 1.69 0.09 1.55 1.82 2720 1

Figure 4. Posterior probability distributions from the new model (left) and old model (right)


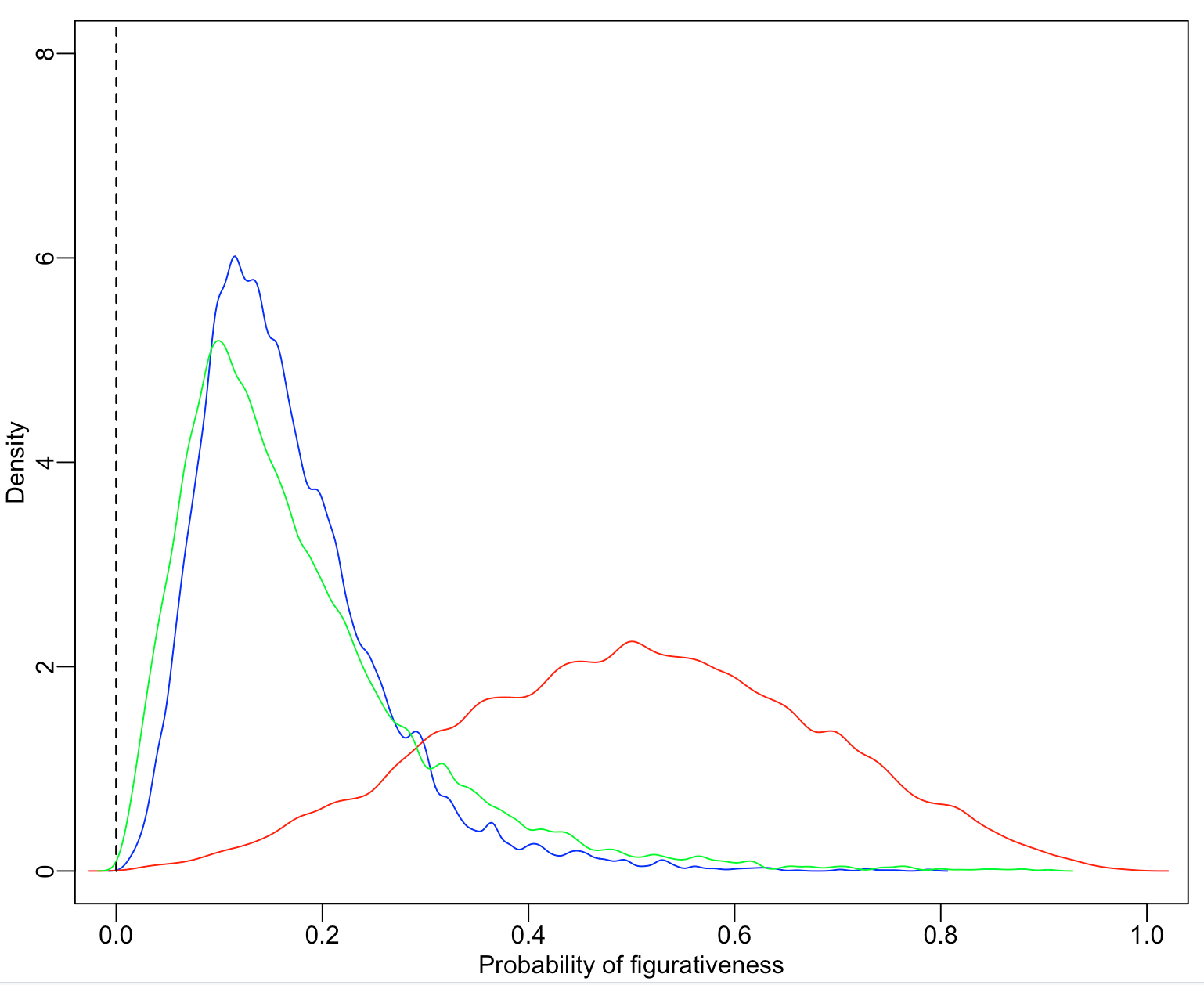

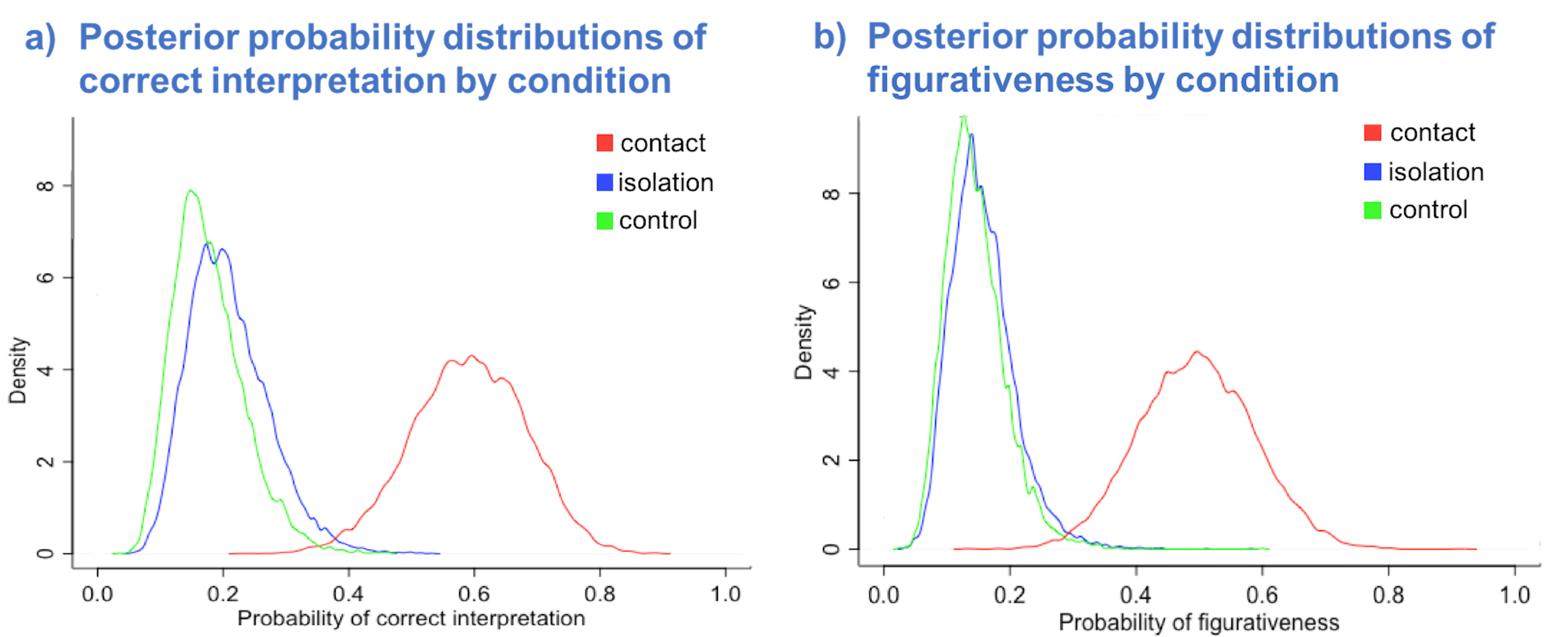


## Frequentist models

Beside the Bayesian models presented in the main text, here we provide the equivalent models run with the traditional frequentist approach. The code is available upon reasonable request.

To estimate the effect of the experimental conditions on the transparency of drawings and their style of representation, response accuracy and figurativeness were analysed by item using logistic regression models fitted with generalised linear mixed-effects regression with a binomial family and a logit link (lme4 package, Bates, Maechler, Bolker, & Walker, 2015; R version 3.3.2, R Core Team, 2016). Condition was introduced as a fixed factor; we included as covariates or random factors the other variables that might account for some variance in the data (see below).

To compare our full models to reduced models including only the random factors and excluding the fixed factor, we compared their AIC values.

The significance of predictors’ coefficients (p values) was obtained from their Z-distributed ratio to their standard errors. The effect sizes of significant coefficients were estimated with their odds ratio, reported as OR (how many times greater a drawing's odds of being correctly interpreted/being figurative are, when varying the predictor, Field, Miles, & Field, 2012; for a published example, Chudek, Heller, Birch, & Henrich, 2012). Note that in R the summary of fixed effects returns tests based on a factor's contrasts, comparing all levels to a baseline level, which we set at isolation for ease of interpretation.

***Are drawings from the contact condition more likely to be interpreted correctly than drawings from the isolation and control condition?***

Yes. Our analysis regressed binary response accuracy on (1) the fixed factor condition (i.e. whether the drawing had been produced in the contact, isolation, or control conditions), (2) the covariate kind of concept (i.e. whether the drawing represented an abstract or concrete concept), (3) the covariate list of concepts (i.e. list A or list B). We specified as a random factor the concept represented in the drawing (N=24); we also specified questionnaire as a random factor (N=18) since – for practical necessities – drawings were sorted in different questionnaires taken by different sets of participants. The model was corrected for overdispersion by introducing an item-level random factor (Browne, Subramanian, Jones, & Goldstein, 2005; Harrison, 2014).

This model had a lower AIC than the corresponding reduced model, which indicated a better fit (AIC_full_ =2692.6, AIC_reduced_=2729.6).

As shown in Supplementary Table 1, in this model there was a significant effect of the contact condition against the baseline category isolation. Specifically, the odds of a drawing being correctly interpreted were 5.73 times greater if it came from the contact rather than the isolation condition. Multiple pairwise comparisons revealed that the contact condition also significantly differed from the control condition, where the odds of a drawing being interpreted correctly were much lower: the odds ratio for correct interpretation of control over contact drawings were 0.143. The odds of correct interpretation of control and isolation drawings did not significantly differ. Response accuracy was not confounded by the covariates kind of concept (p=.226) or list (p=.759).

***Are the drawings from the contact condition more likely to be figurative than the drawings from the isolation and control condition?***

Yes. Our analysis regressed figurativeness on (1) the fixed factor condition (i.e. whether the drawing had been produced in the contact, isolation, or control experimental conditions), (2) the covariate kind of concept (i.e. whether the drawing represented an abstract or concrete concept), (3) the covariate list of concepts (i.e. to which of the two lists used in the Pictionary game the represented concept belonged). We specified the concept represented in the drawing as a random factor (N=24). The model was corrected for overdispersion by introducing an item-level random factor (Harrison 2014, Browne 2005).

This model had a lower AIC than the corresponding reduced model, which indicated a better fit (AIC_full_ = 2465.8, AIC_reduced_= 2568.5).

As shown in Supplementary Table 1, in this model there was a significant effect of condition on figurativeness. Contact condition was different from isolation condition (p<.001); specifically, the odds of a drawing being figurative were 5.48 times greater if it came from the contact rather than the isolation condition (CI_.95=_[3.768 - 8.043], p<.001). Multiple pairwise comparisons revealed that the contact condition also differed from the control condition, where the odds of a drawing being figurative were much lower: the odds ratio for being figurative of control over contact drawings were .166 (CI_.95_=[0.106 - 0.260], p<.001). Finally, the odds of a drawing being figurative did not differ between control and isolation conditions (CI_.95_=[0.615 - 1.344], p=.630.).

Although not related to our hypotheses, we also report an effect of kind of concept, with concrete concepts having significantly lower odds of being figurative than abstract concepts (OR=.360, CI_.95_=[0.173 - 0.736], p=.004). Style category was not confounded by the covariate list of concepts (p=.345).

Overall, the pattern of results of the frequentist models mirrors the pattern of results of the Bayesian models shown in the main text.

# Supplementary Tables

| **Models** | **Predictors** | **Coefficients** | **SE** | **z-values** | **Coefficients CI** | **Odds Ratios** | **Odds Ratios CI** |
| --- | --- | --- | --- | --- | --- | --- | --- |
| Transparency model | Condition is Contact | 1.745*** | .161 | 10.804 | 1.409, 2.088 | 5.726 | 4.093, 8.069 |
|  | Condition is Control | - .201 | .162 | -1.241 | - .545, .137 | .818 | .580, 1.147 |
|  | Kind of Concept is Concrete | - .458 | .378 | 1.210 | - .318, 1.235 | 1.581 | .727, 3.438 |
|  | List of Concepts is B | .119 | .387 | -0.306 | - .912, .671 | .888 | .402, 1.956 |
|  | N | 648 |  | | | | |
| Style model | Condition is Contact | 1.701*** | .193 | 8.820 | 1.326, 2.085 | 5.479 | 3.768, 8.043 |
|  | Condition is Control | - .096 | .199 | -0.482 | -.486, 296 | .909 | .615, 1.344 |
|  | Kind of Concept is Concrete | -1.021** | .354 | -2.886 | -1.755, -.307 | .360 | .173, .736 |
|  | List of Concepts is B | - .334 | .353 | -0.947 | -1.063, .384 | .716 | .345, 1.468 |
|  | N | 648 |  | | | | |

Supplementary table S1 - Summary of the frequentist models

Logistic regression coefficients and their standard errors, 95% CI, Odds Ratios and their 95% CI. The Transparency model regresses response accuracy onto the listed predictors, whereas the Style model regresses figurativeness onto the listed predictors. Condition encodes whether a drawing came from the contact, isolation or control experimental conditions, the baseline in this model being isolation; Kind of Concept encodes whether the drawing represents an abstract or a concrete concept; List of Concepts encodes whether a drawing is representing a concept coming from list 1 or 2. N is the number of observations on which the statistical inference was based.

Supplementary table S2 - Pair composition over the 36 rounds of the isolation condition

|  | **Group 1** | **Group 2** | **Group 3** |
| --- | --- | --- | --- |
| Home Block 1 | | | |
| Game 1 | A-B | D-E | G-H |
| Game 2 | C-A | F-D | I-G |
| Game 3 | B-C | E-F | H-I |
| Game 4 | A-C | D-F | G-I |
| Game 5 | B-A | E-D | H-G |
| Game 6 | C-B | F-E | I-H |
| Home Block 2 | | | |
| Game 7 | B-C | E-F | H-I |
| Game 8 | A-B | D-E | G-H |
| Game 9 | C-A | F-D | I-G |
| Game 10 | B-A | E-D | H-G |
| Game 11 | C-B | F-E | I-H |
| Game 12 | A-C | D-F | G-I |
| Home Block 3 | | | |
| Game 13 | C-A | F-D | I-G |
| Game 14 | B-C | E-F | H-I |
| Game 15 | A-B | D-E | G-H |
| Game 16 | C-B | F-E | I-H |
| Game 17 | A-C | D-F | G-I |
| Game 18 | B-A | E-D | H-G |
| Home Block 4 | | | |
| Game 19 | A-C | D-E | G-H |
| Game 20 | B-A | F-D | I-G |
| Game 21 | C-B | E-F | H-I |
| Game 22 | A-B | D-F | G-I |
| Game 23 | C-A | E-D | H-G |
| Game 24 | B-C | F-E | I-H |
| Home Block 5 | | | |
| Game 25 | B-A | E-F | H-I |
| Game 26 | C-B | D-E | G-H |
| Game 27 | A-C | F-D | I-G |
| Game 28 | B-C | E-D | H-G |
| Game 29 | A-B | F-E | I-H |
| Game 30 | C-A | D-F | G-I |
| Home Block 6 | | | |
| Game 31 | C-B | F-E | I-H |
| Game 32 | A-C | D-F | G-I |
| Game 33 | B-A | E-D | H-G |
| Game 34 | C-A | F-D | I-G |
| Game 35 | B-C | E-F | H-I |
| Game 36 | A-B | D-E | G-H |
| Final stage: Individual drawing | | | |

Supplementary table S3 - Pair composition over the 36 rounds of the contact condition

|  | **Group 1** | **Group 2** | **Group 3** |
| --- | --- | --- | --- |
| Home Block 1 | | | |
| Game 1 | A-B | D-E | G-H |
| Game 2 | C-A | F-D | I-G |
| Game 3 | B-C | E-F | H-I |
| Game 4 | A-C | D-F | G-I |
| Game 5 | B-A | E-D | H-G |
| Game 6 | C-B | F-E | I-H |
| Travel Block 1 | | | |
| Game 1 | B-D | H-A | F-G |
| Game 2 | I-B | E-H | C-F |
| Game 3 | D-I | A-E | G-C |
| Game 4 | B-I | H-E | F-C |
| Game 5 | D-B | A-H | G-F |
| Game 6 | I-D | E-A | C-G |
| Home Block 2 | | | |
| Game 1 | B-C | E-F | H-I |
| Game 2 | A-B | D-E | G-H |
| Game 3 | C-A | F-D | I-G |
| Game 4 | B-A | E-D | H-G |
| Game 5 | C-B | F-E | I-H |
| Game 6 | A-C | D-F | G-I |
| Travel Block 2 | | | |
| Game 1 | C-H | I-F | E-B |
| Game 2 | D-C | A-I | G-E |
| Game 3 | H-D | F-A | B-G |
| Game 4 | C-D | I-A | E-G |
| Game 5 | H-C | F-I | B-E |
| Game 6 | D-H | A-F | G-B |
| Home Block 3 | | | |
| Game 1 | C-A | F-D | I-G |
| Game 2 | B-C | E-F | H-I |
| Game 3 | A-B | D-E | G-H |
| Game 4 | C-B | F-E | I-H |
| Game 5 | A-C | D-F | G-I |
| Game 6 | B-A | E-D | H-G |
| Travel Block 3 | | | |
| Game 1 | A-G | E-C | H-F |
| Game 2 | D-A | I-E | B-H |
| Game 3 | G-D | C-I | F-B |
| Game 4 | A-D | E-I | H-B |
| Game 5 | G-A | C-E | F-H |
| Game 6 | D-G | I-C | B-F |
| Final stage: Individual drawing | | | |

Supplementary table S4 - Pair composition over the 36 rounds of the control condition

|  | **Pairs** |
| --- | --- |
| Home Block 1 | |
| Game 1 | H-C |
| Game 2 | C-F |
| Game 3 | B-C |
| Game 4 | B-F |
| Game 5 | E-G |
| Game 6 | C-D |
| Home Block 2 | |
| Game 7 | A-D |
| Game 8 | B-E |
| Game 9 | H-A |
| Game 10 | D-G |
| Game 11 | I-B |
| Game 12 | A-C |
| Home Block 3 | |
| Game 13 | B-D |
| Game 14 | F-H |
| Game 15 | G-H |
| Game 16 | G-A |
| Game 17 | C-G |
| Game 18 | E-H |
| Home Block 4 | |
| Game 19 | F-G |
| Game 20 | E-I |
| Game 21 | F-I |
| Game 22 | I-C |
| Game 23 | E-F |
| Game 24 | H-I |
| Home Block 5 | |
| Game 25 | G-B |
| Game 26 | G-I |
| Game 27 | A-B |
| Game 28 | D-F |
| Game 29 | A-E |
| Game 30 | I-A |
| Home Block 6 | |
| Game 31 | I-D |
| Game 32 | D-E |
| Game 33 | D-H |
| Game 34 | C-E |
| Game 35 | F-A |
| Game 36 | H-B |
| Final stage: individual drawing | |
